# Subcutaneous edema segmentation on abdominal CT using multi-class labels and iterative annotation

**DOI:** 10.1007/s11548-024-03262-4

**Published:** 2024-09-14

**Authors:** Sayantan Bhadra, Jianfei Liu, Ronald M. Summers

**Affiliations:** Imaging Biomarkers and Computer-Aided Diagnosis Laboratory, Clinical Center, National Institutes of Health, Bethesda, 20892 Maryland USA

**Keywords:** Anasarca, Edema segmentation, nnU-Net, Weakly supervised learning, Iterative annotation

## Abstract

****Purpose**:**

Anasarca is a condition that results from organ dysfunctions, such as heart, kidney, or liver failure, characterized by the presence of edema throughout the body. The quantification of accumulated edema may have potential clinical benefits. This work focuses on accurately estimating the amount of edema non-invasively using abdominal CT scans, with minimal false positives. However, edema segmentation is challenging due to the complex appearance of edema and the lack of manually annotated volumes.

****Methods**:**

We propose a weakly supervised approach for edema segmentation using initial edema labels from the current state-of-the-art method for edema segmentation (Intensity Prior), along with labels of surrounding tissues as anatomical priors. A multi-class 3D nnU-Net was employed as the segmentation network, and training was performed using an iterative annotation workflow.

****Results**:**

We evaluated segmentation accuracy on a test set of 25 patients with edema. The average Dice Similarity Coefficient of the proposed method was similar to Intensity Prior (61.5% vs. 61.7%; $p=0.83$). However, the proposed method reduced the average False Positive Rate significantly, from 1.8% to 1.1% ($p<0.001$). Edema volumes computed using automated segmentation had a strong correlation with manual annotation ($R^2=0.87$).

****Conclusion**:**

Weakly supervised learning using 3D multi-class labels and iterative annotation is an efficient way to perform high-quality edema segmentation with minimal false positives. Automated edema segmentation can produce edema volume estimates that are highly correlated with manual annotation. The proposed approach is promising for clinical applications to monitor anasarca using estimated edema volumes.

## Introduction

**Fig. 1:**
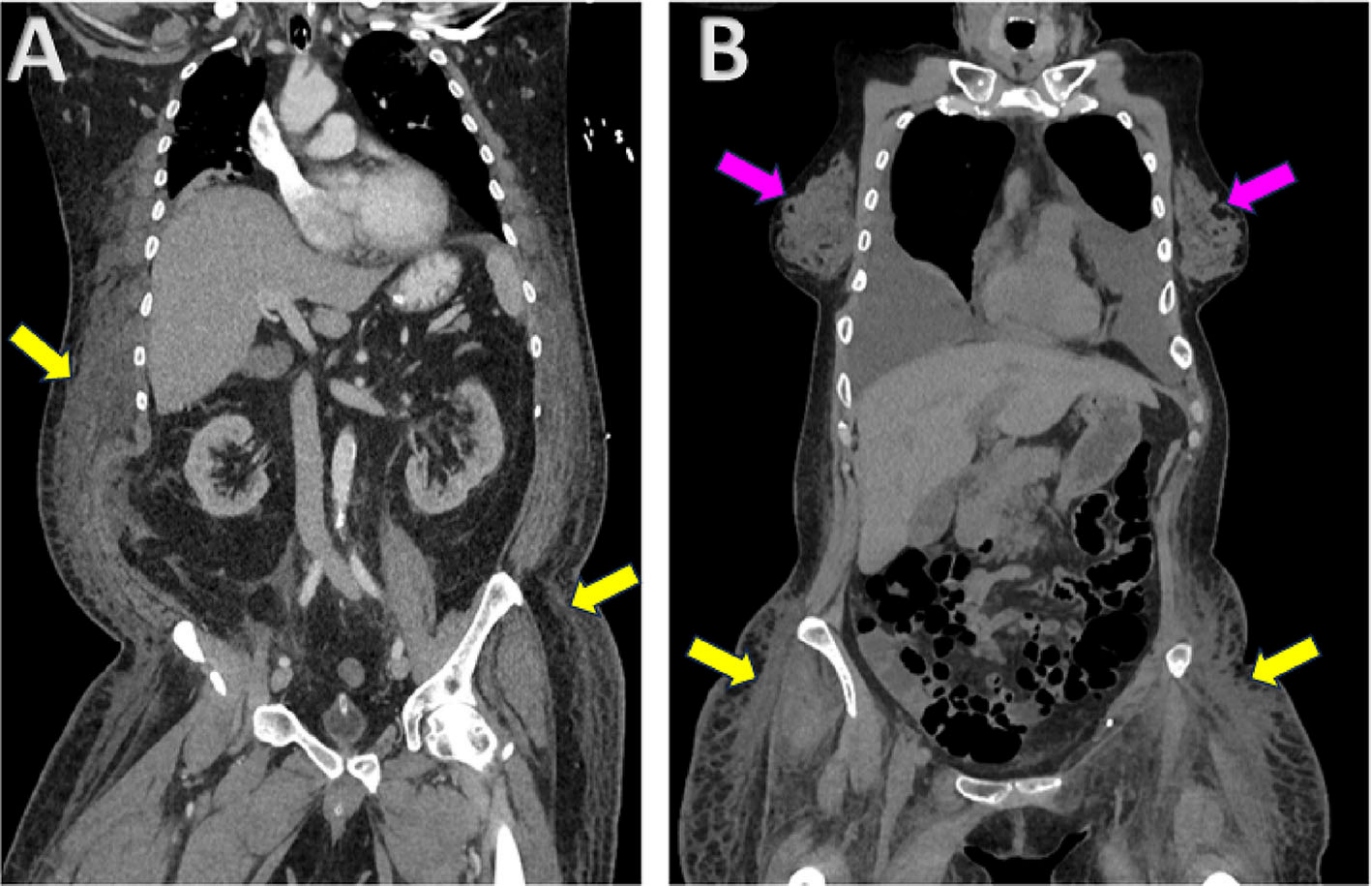
CT scans of male (**A**) and female patients (**B**), with edema present throughout the subcutaneous adipose tissue (yellow arrows). While edema has visible intensity differences with surrounding adipose tissue, it has complex shapes, unclear boundaries, and similar intensity characteristics with other tissues, e.g., glandular breast tissue in women (magenta arrows)

Anasarca is the condition of excessive accumulation of edema throughout the body [1, 2]. Anasarca typically indicates organ dysfunctions, such as kidney disease [3], heart failure [4], and liver cirrhosis [5]. Accurate quantification of edema arising due to anasarca can be potentially useful for monitoring the progression of such organ dysfunctions. Coincidentally, patients undergoing treatment for these diseases have routine CT scans, which enables a direct and non-invasive way to determine edema volume using image segmentation methods [6, 7]. However, the task of edema segmentation is particularly challenging, primarily because edema is diffused within the adipose tissue throughout the patient’s body, characterized by unclear boundaries with surrounding tissues, varying intensity distribution, and irregular shapes (Fig. 1).

Due to its complex appearance, it is extremely challenging to manually annotate edema volumes in CT scans of anasarca patients. This has prohibited the use of supervised deep learning methods for edema segmentation [6, 7]. The current state-of-the-art method for edema segmentation [7] performs unsupervised deep learning by modeling the difference in intensity distributions of edema and surrounding adipose tissue within a level set framework [8, 9]. The intensity distributions are computed within a conditionally generated 2D adipose tissue mask [10]. This method will be referred to as the Intensity Prior method throughout the paper. The Intensity Prior method enables identifying all possible regions with the presence of edema, but at the cost of including false positives with a similar intensity, such as glandular breast tissue (Fig. 1).

Weakly supervised semantic segmentation is an efficient way to refine weak annotations, mitigate false positives, and generate more accurate segmentation labels [11, 12]. In this paper, we propose to improve the segmentation accuracy of subcutaneous edema by utilizing labels from the Intensity Prior method as initial annotations within a weakly supervised learning framework. Additionally, we use labels of tissues surrounding edema to act as anatomical priors [13–15] and reduce the possibility of noisy false positive edema labels. Our method relies on the 3D nnU-Net [16, 17] as the multi-class segmentation backbone, along with iterative refinement of intermediate pseudo-labels [18, 19]. The proposed weakly supervised learning approach reduced false positive edema labels significantly and may enable accurate volumetric measurements of edema for monitoring anasarca.

**Fig. 2:**
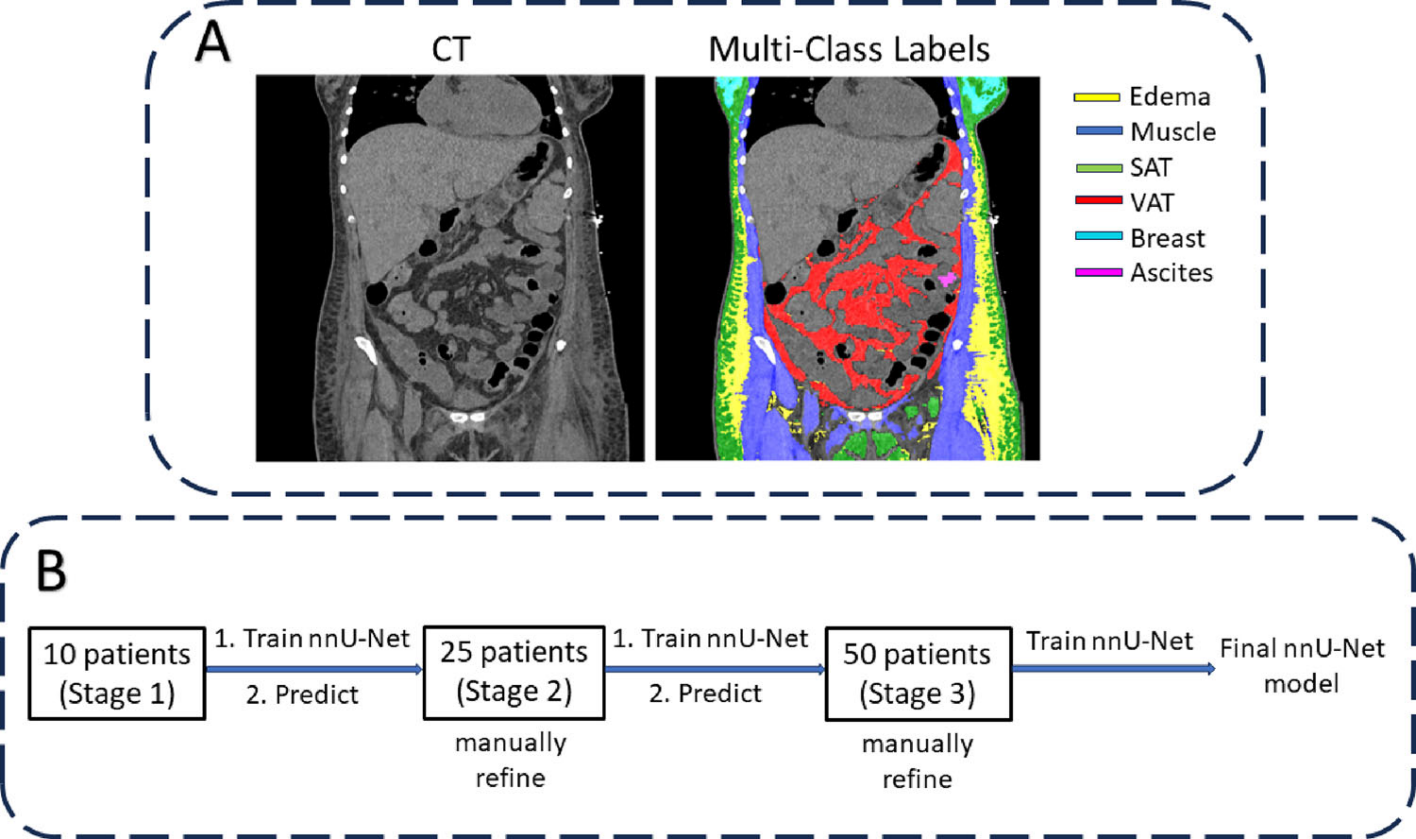
Schematic of the proposed weakly supervised method for edema segmentation: **A** Initial multi-class labels on an abdominal CT scan of a female patient for training the nnU-Net and **B** the iterative annotation workflow

## Methods

### Generation of multi-class labels for training nnU-Net

Our multi-class segmentation model was trained using initial labels of edema derived using the Intensity Prior method, as well as labels of muscle and fat (subcutaneous and visceral) tissues [20, 21] as anatomical priors. To minimize false positive edema labels in glandular breast tissues of women, or due to fluid accumulation from ascites [22] in some anasarca patients, we also added corresponding labels as anatomical priors. We obtained initial weak labels for the breast tissue by manually creating an approximate 3D bounding box region that included the breast region and assigning false positive edema labels produced by Intensity Prior within the bounding box as breast labels. The labels for ascites were computed using a pre-trained 3D nnU-Net that produces accurate segmentations for ascites [23].

**Fig. 3:**
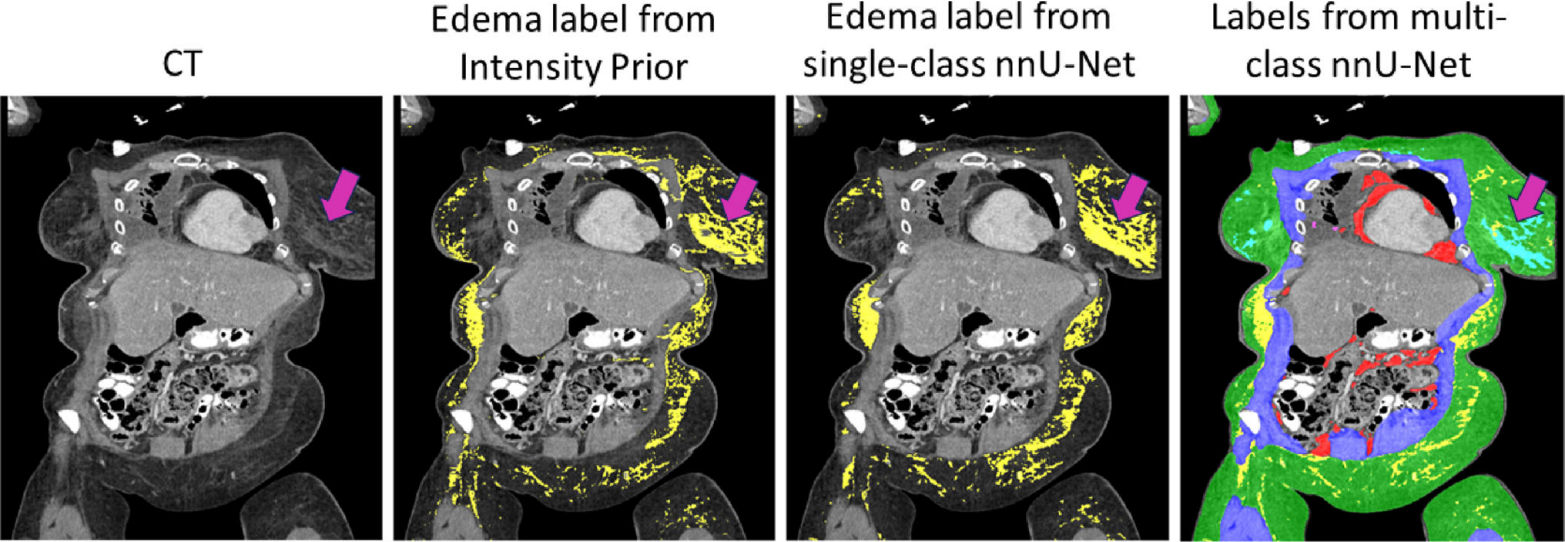
Comparison of labels from Intensity Prior, a single-class nnU-Net and a multi-class nnU-Net. The presence of labels other than edema (yellow) in the multi-class model, such as for glandular breast tissue (cyan), mitigates false positives that otherwise appear with the Intensity Prior method and the single-class nnU-Net model

The motivation for using multi-class labels instead of only the edema class for training the nnU-Net was to provide the context of regions surrounding edema diffused in subcutaneous adipose tissue (SAT) with unclear boundaries and potentially improve the segmentation accuracy of edema [14, 15]. Figure 3 compares segmentations from the Intensity Prior method, a single-class nnU-Net model trained on edema labels from the Intensity Prior method, and a multi-class nnU-Net model. The single-class model produces large amounts of false positive edema labels in the breast tissue due to the absence of the breast label as an anatomical prior, as opposed to the multi-class model. Additionally, the presence of multi-class labels delineating different tissues with similar intensities is visually useful for manual refinement of edema labels during iterative annotation, which will be described next.

### Iterative annotation of edema labels

Our weakly supervised segmentation approach consisted of multiple stages of nnU-Net training with different amounts of training data in an iterative learning framework [18]. The iterative process involved sequentially training the nnU-Net and predicting multi-class labels on unlabeled CT images using the trained nnU-Net, which are then manually refined to partially correct mislabeled edema. It should be noted that due to the diffused nature of edema, it was infeasible to manually correct edema in all regions of the CT volume. This was followed by re-training the nnU-Net from scratch with the training dataset augmented using the new CT scans and corresponding pseudo-labels. The iterative annotation workflow is shown in Fig. 2B.

**Fig. 4:**
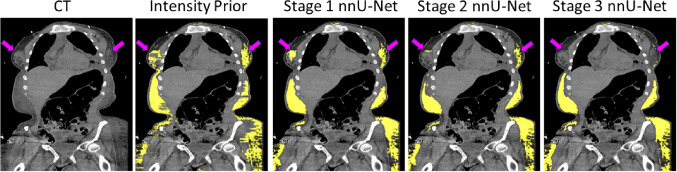
Visual example of the improvement in edema segmentation at different stages of iterative annotation (Fig. 2B). False positives in the breast glandular tissue are progressively removed by re-training the nnU-Net with additional manually refined pseudo-labels

Figure 4 shows a visual example of the improvement in edema segmentation due to iterative annotation. At the end of Stage 1 training with 10 patients and initial multi-class labels that included edema labels from the Intensity Prior method, the nnU-Net produces small amounts of false positives in the breast tissue. These errors are significantly reduced in Stage 2 after the next round of nnU-Net training with additional manually refined pseudo-labels, using a total of 25 patients. However, some errors persist, and these errors are almost completely removed after Stage 3 of training the multi-class nnU-Net using CT scans from 50 patients.

## CT data and validation methods

We trained the proposed multi-class nnU-Net model on a dataset of abdominal CT scans with edema (*n* = 50) from the NIH Clinical Center. The CT scans were collected from 24 female and 26 male patients. The dataset consisted of 21 contrast-enhanced and 29 non-contrast CT scans with a slice thickness of 2 mm. The scans were obtained at 120 kVp on Siemens scanners and with a soft kernel reconstruction algorithm. The presence of edema was confirmed using radiology reports and verified by an experienced radiologist. We performed fivefold cross-validation for training the nnU-Net at each stage of the iterative annotation process. For validation, we used a held-out test dataset of anasarca patients (*n* = 25) from the NIH Clinical Center as utilized in [7]. Reference annotations were available for 5 slices from each test CT scan as described in [7]. We evaluated segmentation accuracy using two metrics: Dice Similarity Coefficient (DSC) and False Positive Rate (FPR). The FPR metric was computed as the total volume of true edema label misclassified as background divided by the total volume inside the body region mask generated using TotalSegmentator [18]. We also computed $R^2$ correlation values of the total edema volume over 5 slices against the manually annotated volume. The baselines for our studies were the Intensity Prior method in [20] and a previous method based on Gaussian Mixture Models (GMM) [6]. Paired sample t-tests were performed to estimate the statistical significance of metrics achieved by the multi-class nnU-Net against each of the other two baselines. All the metrics and statistical tests were computed using the SciPy library (version 1.11.1) [24].

**Fig. 5:**
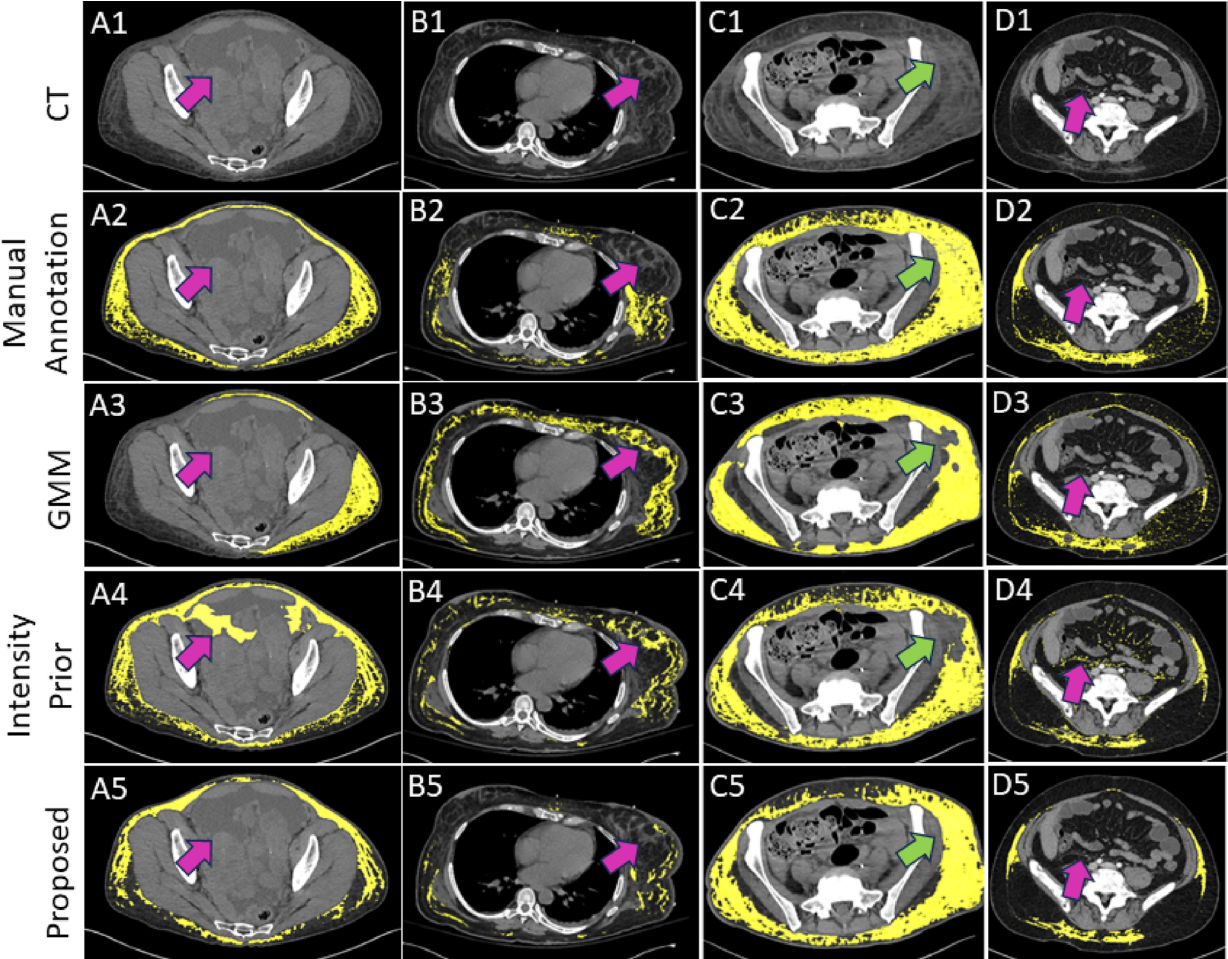
Examples of edema segmentation produced by GMM, Intensity Prior method, and the proposed method on four anasarca patients. Each column (A–D) corresponds to a unique patient. The Intensity Prior method and the proposed multi-class nnU-Net method produce accurate segmentations generally. However, the Intensity Prior method generated visible regions of incorrect segmentation that are mitigated by the proposed method

## Results

Figure 5 shows visual examples of edema segmentations produced by GMM, Intensity Prior, and the proposed method on four patients with edema. Figure 5A–C corresponds to non-contrast CT scans while Fig. 5D is derived from a contrast-enhanced CT scan. In general, we observed that both the Intensity Prior method and the proposed method produced edema labels that were more accurate than GMM when compared with manual annotation. However, the proposed method using multi-class nnU-Net generated edema labels that were more aligned with regions manually annotated as edema. For example, in Fig. 5A4 and B4, we can observe false positive edema labels produced by the Intensity Prior method in regions of ascites fluid and glandular breast tissue, respectively, that were mitigated by the proposed method (Fig. 5A5 and B5). In Fig. 5C, there is a visible region of under-segmentation of edema by the GMM and Intensity Prior methods which is accurately segmented by the proposed method. The multi-class nnU-Net model is also able to correctly segment edema in a contrast-enhanced CT scan in Fig. 5D5, while the Intensity Prior method produced scattered false positive edema labels outside the subcutaneous adipose tissue region (Fig. 5D4).

**Fig. 6:**
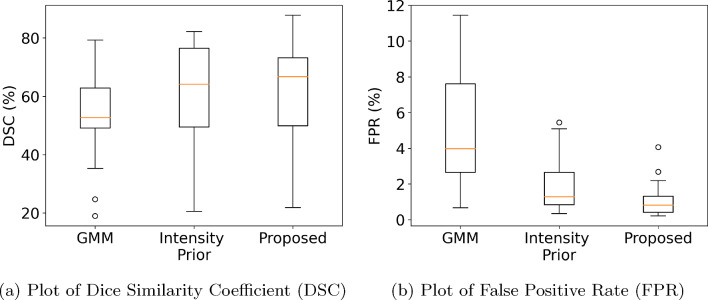
Comparison of **a** Dice Similarity Coefficient (DSC) and **b** False Positive Rate (FPR) metrics for edema segmentation. While the DSC metric could not capture differences in segmentation quality, the FPR metric distinguished the proposed method as the one to produce the lowest amount of false positive segmentations

**Fig. 7:**
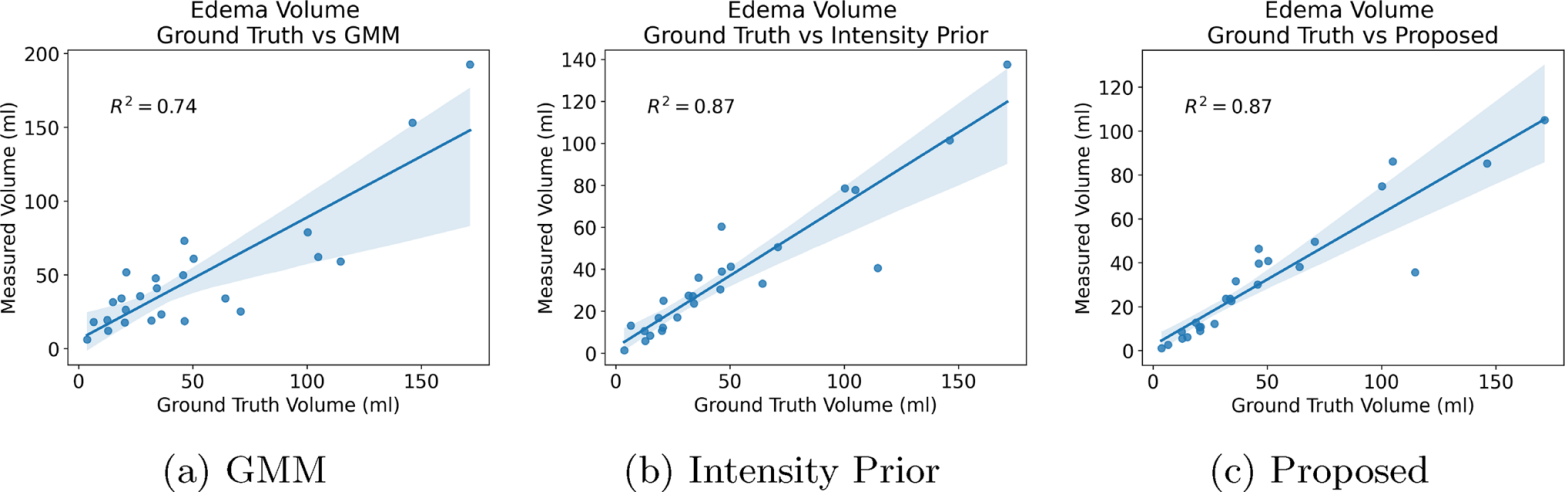
$R^2$ correlation plots of the automated edema segmentation methods compared against manual annotations. All three methods showed a strong correlation, with the Intensity Prior and proposed methods exhibiting a higher correlation ($R^2=0.87$) compared to GMM ($R^2=0.74$)

For a quantitative comparison of segmentation accuracy, we computed the DSC and FPR metrics for all three methods in Table 1, along with corresponding box plots in Fig. 6. Based on the DSC metric, the proposed method did not demonstrate any significant difference compared to GMM ($p=0.13$) or the Intensity Prior method ($p=0.83$). However, with the FPR metric, the multi-class nnU-Net method exhibited significant improvement in reducing false positive edema labels compared to both the GMM and Intensity Prior methods ($p < 0.001$), which aligns with our qualitative observations in Fig. 5. This is an example of the limitations of overlap-based metrics such as DSC in estimating the segmentation accuracy of complex targets like subcutaneous edema [25, 26]. We also computed $R^2$ correlation plots of total edema volume over 5 slices for the three automated segmentation methods as shown in Fig. 7. All three methods demonstrated a strong correlation with edema volumes obtained using manual annotations. The Intensity Prior and proposed methods achieved a higher correlation ($R^2=0.87$) compared to the GMM method ($R^2=0.74$).

## Discussion and conclusion

In this paper, we proposed a weakly supervised learning method for subcutaneous edema segmentation using a multi-class 3D nnU-Net and iterative annotation. The proposed method utilizes initial edema labels from the unsupervised Intensity Prior method [7], along with additional weak and strong labels of surrounding tissues. The presence of multi-class labels and iterative learning using a 3D nnU-Net gradually mitigated false positives and improved segmentation accuracy compared to the state-of-the-art Intensity Prior method. Quantitatively, while the average Dice Similarity Coefficient remained similar to the Intensity Prior method, the average False Positive Rate reduced significantly, from 1.8 to 1.1%. Regression plots of edema volumes estimated using automated methods showed a strong correlation with manual annotation.

**Table 1:** Comparison of segmentation accuracy between GMM, Intensity Prior, and the proposed method on 25 abdominal CT scans with edema

Metric	GMM	Intensity Prior	Proposed
DSC (%) ($\uparrow$)	$53.8 \pm 14.0$	$61.7 \pm 17.6$	$61.5 \pm 17.9$
FPR (%) ($\downarrow$)	$5.0 \pm 3.0$	$1.8 \pm 1.4$	$\mathbf {1.1 \pm 0.9}$

One of the limitations of our work is that segmentation accuracy was validated on a test set of only 25 CT scans collected from a single institution, with 5 slices annotated in each CT volume. The main reason for this limitation is that manual annotation of edema is extremely time-consuming, even for expert radiologists, resulting in a small number of reference annotation slices. Manual annotation of edema may also have high uncertainty due to its subjective nature, as noted in inter-observer studies [7]. Additionally, it is challenging to perform external validation on anasarca patients from other institutions due to the lack of curated publicly available datasets of anasarca patients.

The ability to perform automatic and accurate segmentation of subcutaneous edema can enable potential clinical applications for monitoring anasarca using measured edema volumes. Currently, body weight measurements are recorded to monitor fluid balance of patients in the intensive care unit (ICU), which may not be reliable [27]. The availability of objective ways to measure edema volume using CT imaging can lead to new directions of monitoring fluid balance during the treatment of anasarca patients.

While our studies focused on subcutaneous edema, anasarca patients also exhibit varying amounts of visceral edema [28, 29], as well as pleural and pericardial effusions [30, 31]. Future work would involve developing a single model capable of segmenting different types of fluids that co-occur with edema in anasarca patients.
